# Wound repair and immune function in the *Pseudomonas* infected CF lung: before and after highly effective modulator therapy

**DOI:** 10.3389/fcimb.2025.1566495

**Published:** 2025-04-28

**Authors:** Emma Lea Matthews, Meghan June Hirsch, Federico Prokopczuk, Luke I. Jones, Eriel Martínez, Jarrod W. Barnes, Stefanie Krick

**Affiliations:** ^1^ Division of Pulmonary, Allergy, and Critical Care Medicine, Department of Medicine, The University of Alabama at Birmingham, Birmingham, AL, United States; ^2^ Gregory Fleming James Cystic Fibrosis Research Center, The University of Alabama at Birmingham, Birmingham, AL, United States; ^3^ Department of Microbiology, The University of Alabama at Birmingham, Birmingham, AL, United States

**Keywords:** wound repair, *Pseudomonas aeruginosa*, cystic fibrosis, bronchial epithelium, immune system, modulator therapy, elexacaftor/tezacaftor/ivacaftor(ETI)

## Abstract

The leading cause of death for people with cystic fibrosis (pwCF) continues to be due to respiratory-related illnesses. Both wound repair and immune cell responses are dysregulated in the CF airways, creating a cycle of unresolved injury and perpetuating inflammation. PwCF are predisposed to colonization and infections with opportunistic bacteria like *Pseudomonas aeruginosa (Pa)*, the most common adult pathogen in CF. *Pa* possesses key virulence factors that can exacerbate chronic inflammation and lung injury. With the approval of highly effective modulator therapies like elexacaftor/tezacaftor/ivacaftor (ETI), pwCF eligible for ETI have seen drastic improvements in lung function and clinical outcomes, including an increased life expectancy. While modulator therapies are improving bronchial epithelial cellular processes in wound repair and some areas of immunity, many of these processes do not reach a non-CF baseline state or have not been thoroughly studied. The effect of modulator therapy on *Pa* may lead to a reduction in infection, but in more longitudinal studies, there is not always eradication of *Pa*, and colonization and infection frequency can return to pre-modulator levels over time. Finally, in this review we explore the current state of additional treatments for CF lung disease, independent of CFTR genotype, including anti-inflammatories, phage-therapies, and *Pa* vaccines.

## 1 Introduction

Cystic Fibrosis (CF) is an autosomal recessive disorder that leads to ion imbalance in the epithelium of organs such as the pancreas, gastrointestinal tract, and the lungs (Shteinberg et al., 2021). Mutations in the cystic fibrosis transmembrane conductance regulator (*CFTR*) gene lead to dysfunctional protein, impaired protein trafficking, or in some cases, complete absence of the CFTR protein. Without treatment, CFTR dysfunction causes multisystem complications with significant disease burden and a decreased life (Elborn, 2016). The leading cause of death for people with CF (pwCF) in 2023 was due to respiratory-related illnesses (Cystic Fibrosis Foundation, 2024). The absence or dysfunction of CFTR at the epithelial membrane results in poor mucociliary clearance in the lung along with several other physiological changes that provide a suitable environment for opportunistic infection with pathogens such as *Pseudomonas aeruginosa* (*Pa*) (Elborn, 2016). *Pa* is a gram-negative bacterium that poses a significant threat to pwCF due to its ability to establish chronic lung infections, and such infections strongly correlate with decreased lung function and increased mortality (Lyczak et al., 2002; Bhagirath et al., 2016). *Pa* is the most common lung pathogen of adult pwCF and can produce many virulence factors that cause and exacerbate lung damage, leading to colonization and recurrent infections (Bhagirath et al., 2016; Cystic Fibrosis Foundation, 2024).

Wound repair is a dynamic process with various cellular regulators, many of which are dysfunctional in CF (Hilliard et al., 2007). This dysfunction leads to a delayed repair process, exacerbating already increased inflammation and reducing the CF lung’s ability to combat threats like bacterial infection with *Pa* (Conese and Di Gioia, 2021). At least 50% of pwCF aged 35 or older are expected to develop advanced lung disease, which is characterized by severely impaired lung function and airway remodeling as a result of aberrant wound repair (Conese and Di Gioia, 2021; Cystic Fibrosis Foundation, 2024). Therefore, research to advance the understanding of wound repair mechanisms in the chronically infected CF lung is of high importance to ultimately develop therapeutic approaches to restore these processes.

In addition to the cellular regulators of wound repair and the abnormal properties (e.g. thick mucus) caused by defective CFTR, specific immune cell populations are also negatively affected in CF. Neutrophils and macrophages, in particular, exhibit compromised phagocytic capabilities and inflammatory signaling, leading to chronic infection and inflammation (Hayes et al., 2011; Harwood et al., 2021). Lymphoid cells and eosinophils also play a role in CF pathogenesis, but their contributions remain less studied (Liu et al., 1999; Harwood et al., 2021).

Over the past decade, the development of CFTR modulator therapies which include potentiators, correctors, stabilizers, and amplifiers has significantly improved the prognosis of pwCF (Lopes-Pacheco, 2019). There has been robust improvement in lung pathophysiology in pwCF, particularly by the highly effective modulator combination therapy of elexacaftor/tezacaftor/ivacaftor (ETI) (Lupas et al., 2024). One of the remaining questions that continues to be a source of debate in this field is the long-term effect of CFTR modulators on cellular processes in the CF lung, specifically on wound repair mechanisms, immune cell function, and susceptibility to *Pa* infection.

This review will focus on the elucidation of mechanisms behind CF lung repair in response to 1- injury following infection; 2- virulence strategies employed by *Pa* to damage and impair these processes; and 3- the complex immune system mechanisms to combat *Pa*. Moreover, this review will explore how recent advances in CFTR modulator therapies have affected all these processes and discuss other therapeutic strategies on the horizon for pwCF. We aim to provide a broad overview of wound repair, *Pa* infection, and related immune cell processes in the CF lung prior to modulator therapy in order to consider the implications of ETI on these process and further future directions in understanding the CF lung environment.

## 2 Wound repair mechanisms

### 2.1 Injury repair in the bronchial epithelium

The mature lung has traditionally been viewed as a quiescent organ with minimal regenerative capacity. However, the lung possesses a unique and remarkable capacity to maintain homeostasis despite injury through extensive wound repair mechanisms that are similar to the mechanisms of general wound repair. Pulmonary epithelial wound repair has been reviewed at great length elsewhere (Crosby and Waters, 2010; Croasdell Lucchini et al., 2021); however, we will provide a brief overview here.

In response to an epithelial insult in the lung, an initial acute inflammatory phase results in the activation of tissue resident macrophages followed by the recruitment of additional inflammatory cells to the site of injury (Wynn and Vannella, 2016). Injured epithelial cells as well as the immune cell environment release a variety of soluble mediators and chemokines such as epidermal growth factor, which recruit progenitor cell populations to the site of injury and initiate the wound repair process (Van Winkle et al., 1997; Kim et al., 1998; Croasdell Lucchini et al., 2021). This signaling milieu, moreover, results in the expression of tissue remodeling and extracellular matrix (ECM) proteins such as collagens, fibronectin, and matrix metalloproteinases (MMPs), among others, which aid in the migration and proliferation of epithelial cells about the site of injury (Rickard et al., 1993; Legrand et al., 1999; Coraux et al., 2008; Ojo et al., 2015). Transforming growth factor beta (TGF-β) signaling further plays a vital role in the re-epithelialization process as its initiation results in the epithelial-mesenchymal transition (EMT), which aids in the formation of a provision ECM network across which cell migration and remodeling can occur (Kim et al., 2006; Câmara and Jarai, 2010). Importantly, the EMT is often dysregulated in pathogenesis and has been described in a variety of lung disease states including idiopathic pulmonary fibrosis, pulmonary hypertension, and cancer.

A growing body of evidence suggests that following lung injury, stem cell progenitor populations and/or self-renewing cell types are recruited to reestablish a continuous and functional epithelial surface (Kotton and Morrisey, 2014). One of the greatest challenges to understanding the injury response in the airway epithelium is great redundancy in the regenerative capacity of the many subpopulations within the bronchial epithelium. The basal cell has classically been identified as a pulmonary stem population, particularly in the proximal airways, with the capacity to give rise to an assortment of cell populations including ciliated and goblet cells (Ruysseveldt et al., 2021). The field of progenitor cell biology in the lung is a frontier of novel research as new regenerative cell types are still being discovered (Basil et al., 2022).

### 2.2 Wound repair in the CF lung

Chronic inflammation and aberrant injury repair are constants in the CF lung. Only recently, some of the mechanisms in which this occurs have been elucidated including protease/anti-protease imbalances of neutrophil elastase (NE), MMPs, and other proteases which have been noted in CF pathogenesis, all of which contribute to tissue damage and remodeling (Hilliard et al., 2007) (Fischer et al., 2013). Additionally, molecular markers of injury repair are increased for an extended period, including MMP-9, TGF-β1, fibronectin, vimentin, and integrin β1, and a decrease in E-cadherin (Conese and Di Gioia, 2021). While many of these proteins are associated with EMT, which as discussed previously, is an essential step in healthy injury repair, the CF lung appears to have a prolonged and aberrant EMT state, contributing to further chronic inflammation and injury.

The perpetuating cycle of chronic inflammation found in the CF lung results in the imbalance of pro- and anti-inflammatory mediators, such as increases in interleukin-8 (IL-8) and IL-6, and decreases in IL-10 (Courtney et al., 2004). This pattern of increased inflammation has been shown to play a role in not only damaging the epithelium, but also in dysregulating repair and remodeling (Shute et al., 2003; Courtney et al., 2004; Virella-Lowell et al., 2004; Conese and Di Gioia, 2021).

Cellular senescence is defined by irreversible cell cycle growth arrest, which occurs in response to cellular stressors such as inflammation (Campisi and d’Adda di Fagagna, 2007; Herranz and Gil, 2018). Senescent cells are resistant to apoptosis and acquire a senescence associated secretory phenotype (SASP), which stimulates secretion of pro-inflammatory mediators that perpetuate tissue damage and inflammation (Hernandez-Segura et al., 2018). Transient cellular senescence is a crucial part of normal wound repair, but disease states occur with persistent senescence, which can overwhelm the immune system to effectively clear these cells (Wilkinson and Hardman, 2020). Several studies including one from Easter et al. found that cellular senescence markers are upregulated in the CF bronchial epithelium when compared to non-CF controls at baseline, suggesting another mechanism that could be dysregulating wound repair in the CF bronchial epithelium and one of many reasons for the compounding lung injury that pwCF deal with overtime (Nakamura et al., 1992; Fischer et al., 2013; Easter et al., 2024).

**Figure 1:**
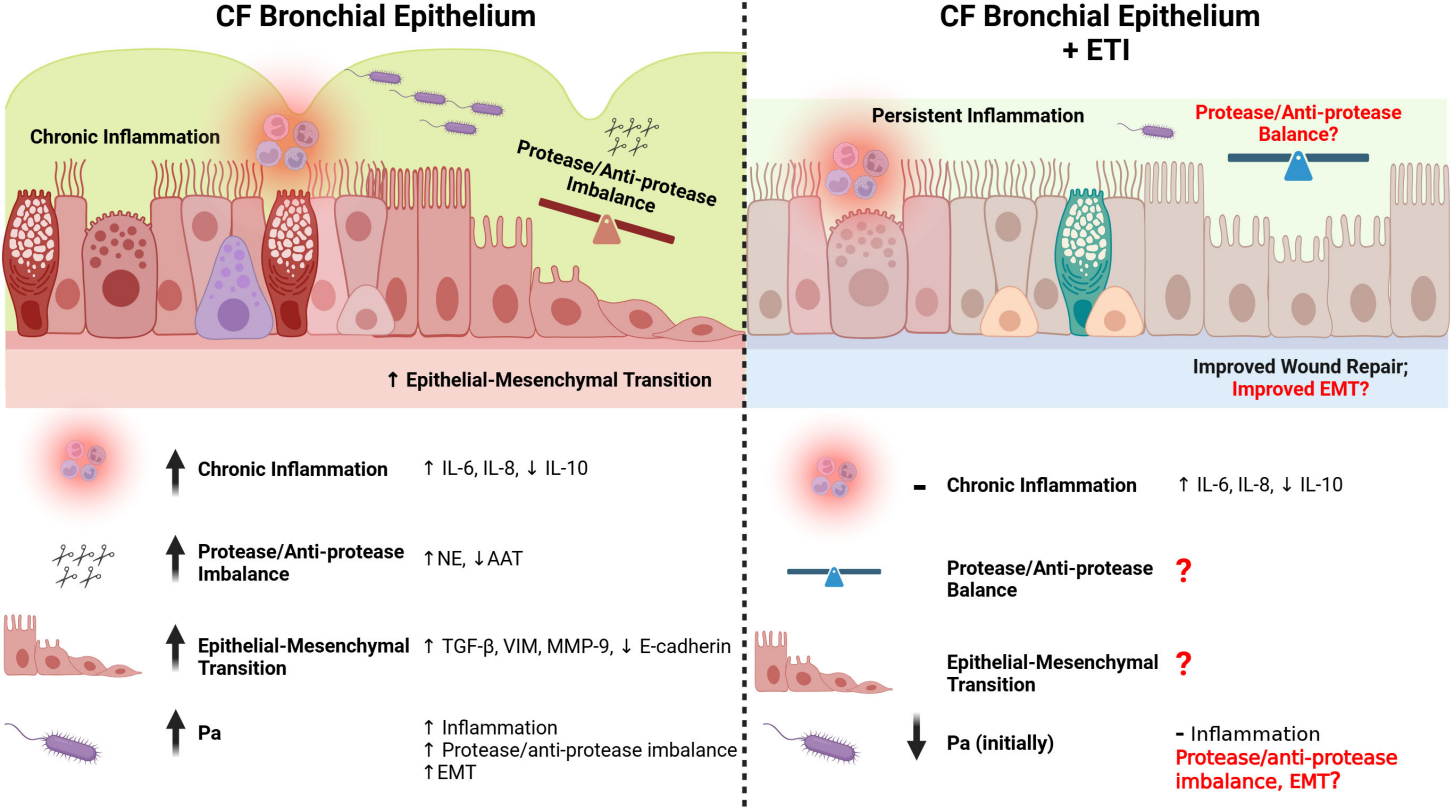
Repair in the CF bronchial epithelium before and after ETI therapy. The CF bronchial epithelium is subject to chronic inflammation, partially as a result of *Pa* infection and a subsequent dysfunctional immune response (see **Figure 2**). Pro-inflammatory cytokines (e.g. IL-6, IL-8) are upregulated in the CF bronchial epithelium, while anti-inflammatory cytokines are downregulated (e.g. IL-10). Even after the initiation of ETI, many markers of chronic inflammation are only slightly improved or are unchanged. The protease/anti-protease ratios are imbalanced in the CF bronchial epithelium, with increased NE and decreased AAT. This imbalance causes weaker cell-cell junctions, and a delayed healing process. The protease/anti-protease axis has not been well explored after initiation of ETI, especially in the context of *Pa* infection. The epithelial-mesenchymal transition (EMT) is prolonged in the CF bronchial epithelium, with greater increases in TGF-β, vimentin, MMP-9, and a decrease in E-cadherin when responding to injury. While ETI causes an improvement in wound repair rates in the CF bronchial epithelium, there have not been studies on the effect of ETI on EMT with or without *Pa* infection. Created in https://BioRender.com.

In summary, many studies have shown evidence for dysregulated wound repair in the CF lung, which can be further complicated by chronic airway infection with *Pa* modulating wound repair in the CF lung even further (**Figure 1**).

## 3 The effects of *Pseudomonas aeruginosa* on wound repair

*Pa* is a gram-negative opportunistic pathogen and a major causative organism in a variety of chronic pneumonia, eye infections, burns, diabetic ulcers, etc. This versatility is due not only to its broad arsenal of virulence factors, but its dynamic production of these factors over time. It’s long been established that *Pa* poorly interacts with intact, healthy, and well polarized epithelium (Tsang et al., 1994; Schwarzer et al., 2016). However, in pwCF, the airway epithelium demonstrates increased tight junction leakage, cell death, senescence, remodeling, and division. These factors increase the capacity for the establishment of *Pa* infection. The prevalence of *Pa* in pwCF <2 years-old is under 30%, but at the age of 25 years and older, greater than 50% of pwCF are infected with *Pa* (Rosenfeld et al., 2003; Foundation, 2021).

*Pa* infections can be separated into either acute or chronic infections. These infections exist on a spectrum, but acute infections are more directly cytotoxic and invasive whereas *Pa* strains in chronic infections tend to be more resistant to treatment, mucoid, and sessile. In acute infections, *Pa* breaches the epithelium and accessing the basolateral side of the cell layer where most cytotoxic factors are delivered. Once colonized, *Pa* will become less cytotoxic and more resistant over time. This transition is marked by increased biofilm production and decreased motility factors (Iwanska et al., 2023), leading to the formation of non-moving, resistant infections (Mahenthiralingam et al., 1994; Ramsey and Wozniak, 2005). This resistant *Pa* state can be disrupted, and shift towards more acute *Pa* phenotypes that can lead to pulmonary exacerbations (Aaron et al., 2004).

Throughout this process, *Pa* employs a multitude of virulence factors that will either induce the damage or impair the repair processes. For the purposes of this review, we will only discuss a few examples of *Pa* virulence factors found to both induce damage and affect wound repair in the lung.

### 3.1 Factors contributing to wound induction

Cell-junction destruction is crucial in *Pa* exacerbating pulmonary symptoms in pwCF, as this destruction allows for cytotoxic products to be released in deeper cell layers. *Pa* rhamnolipids can displace important linker proteins and disrupt the tight junctions at the lung basolateral membrane (Zulianello et al., 2006). There is also a positive correlation between rhamnolipid production and acute infection isolates (Laabei et al., 2014). LecA and LecB have been demonstrated to increase *Pa*-induced alveolar capillary permeability (Chemani et al., 2009). LecB has been shown to be crucial to *Pa*’s ability to loosen cell-substrate adhesion (Thuenauer et al., 2020). Other enzymes such as LasB elastase have been shown to degrade key proteins in the extracellular matrix and basement membrane (Guzzo et al., 1991; Coin et al., 1997).

Once the barrier to the basolateral side has been subverted *Pa* can release substances that lead to direct cytotoxicity, exacerbating cell death and tissue injury. Examples include the Type III secretion system (T3SS) effectors ExoS and ExoT that have been shown to induce apoptosis and cause actin cytoskeleton collapse (Ganesan et al., 1999; Shaver and Hauser, 2004).

Lipopolysaccharide (LPS) can trigger inflammatory responses and pyroptotic cell death, which is highly pro-inflammatory in nature (Schoeniger et al., 2016; Huang et al., 2019). Pyocyanin is a secondary metabolite capable of generating reactive oxygen species (ROS) (O’Malley et al., 2003), and has been detected in large quantities in the sputum of pwCF infected with *Pa*, enough to inhibit ciliary beating and cause toxicity in the *in vitro* respiratory epithelium (Kanthakumar et al., 1993).

### 3.2 Factors of wound repair alteration

Several of these *Pa* virulence factors have also been shown to alter cellular repair rates. LecB, has been shown to impair repair processes through decreasing migration and proliferation of human lung epithelial cells and diminishing cell-cell contacts (Cott et al., 2016). ExoT has also been shown to impair repair in A549 alveolar cell monolayers, secondary to actin cytoskeleton disorganization (Geiser et al., 2001). Pyocyanin inhibits wound repair by inducing premature growth arrest of human diploid fibroblasts, causing the development of characteristics associated with cellular senescence (Muller, 2006).

LasB elastase promotes collagen degradation by inducing the conversion of the inactive precursors of several MMP to active enzymes, thus inhibiting proper collagen placement (de Bentzmann et al., 2000). LasB, as well as some previously described factors, are controlled under quorum sensing (QS) systems. One group demonstrated that inhibiting QS dampened the combined action of LasB and LasA proteases, highlighting an alternative strategy to allow for efficient repair of the airway epithelium despite infection (Ruffin et al., 2016; Ruffin and Brochiero, 2019).

In total, *Pa* has a multitude of virulence factors that can cause lung injury directly and indirectly by dysregulating host injury repair mechanisms. How *Pa* virulence factors directly alter repair mechanisms is still an area that needs more investigation, especially in the context of CF affecting not only the airway epithelium but also impacting the immune system of the lung.

## 4 Immune cells and their effect on lung repair in the CF lung

CFTR dysfunction in selected immune cell populations has been shown to have a negative effect on tissue repair and exacerbates the inflammatory phenotype already prevalent in CF. This section will go into further detail on the altered phenotypes of these cell populations, their role in tissue injury/repair, and their interaction with *Pa*.

### 4.1 Neutrophils

PwCF have higher baseline levels of neutrophil recruitment factors including IL-6, IL-8, and TNF-α, leading to an increase in neutrophil recruitment with disputes on whether this occurs prior to infection or after the first infection (Hayes et al., 2011; Giacalone et al., 2020; Aslanhan et al., 2021). When compared to non-CF neutrophils, CF neutrophils shed less L-selectin and are less responsive to IL-8 due to the downregulation of CXCR2, increased mucus in the bronchial epithelium, and the presence of *Pa*-produced alginate (Hayes et al., 2011; Aslanhan et al., 2021; Laucirica et al., 2022; Hu et al., 2024). These factors lead to a reduction in neutrophil recruitment and their bactericidal effects (Zhang et al., 2021).

During the recruitment process, CF neutrophils exhibit the GRIM (Granule releasing, immunoregulatory, and metabolically active) phenotype (Margaroli et al., 2021; Laucirica et al., 2022), which is characterized by a reduced ability to phagocytose bacteria (Giacalone et al., 2020). This phenotype occurs due to the CFTR defect leading to impaired chloride transport and lack of hypochlorous acid production, a vital component of the antibacterial activity of neutrophils (Cabrini et al., 2020).

GRIM neutrophils have decreased CD16 expression post migration and have increased levels of CD63, which is activated by CF mucus and further increased in the presence of *Pa* infection (Laucirica et al., 2022; Hu et al., 2024). It is also known that GRIM neutrophils exocytose high levels of cathepsin G, MPO, Arg1, and NE, which are parts of the secondary defense that neutrophils display during degranulation (Giacalone et al., 2020). However, when transcription is inhibited, GRIM neutrophils had decreased degranulation with increased levels of bacterial killing compared to baseline (Margaroli et al., 2021).

There is debate on whether ROS, secreted by neutrophils in CF can exacerbate inflammation and injury in the lung. Some reports state that oxidative burst and ROS exacerbate CF-associated inflammatory changes and infection in the CF lung (Cabrini et al., 2020; Aslanhan et al., 2021), while others claim no difference in ROS and C-reactive protein between pwCF and healthy controls (Kelk et al., 2022).

The protease to anti-protease ratio is severely skewed in CF as NE dominates the airway and cleaves anti-proteases such as secretory leukocyte inhibitor (SLPI) and alpha-1 antitrypsin (AAT) (Hayes et al., 2011; Houston et al., 2024). The CFTR protein is an identified substrate for NE along with IL-8 receptor CXCR1, elastin, collagen, Fcγ receptors, and MMP9 (Houston et al., 2024).

Finally, CF neutrophils also have dysregulated cell death pathways with increased levels of the anti-apoptotic marker MCL-1 along with increases in p21 and neutrophil survival regulator proliferating cell nuclear antigen (PCNA) (Chiara et al., 2012). This study implicates these proteins as potential targets for inducing apoptosis in CF neutrophils. Additionally, CF neutrophils do not undergo spontaneous apoptosis due to decreases in Fas and FasL along with TNF-α death receptors (Hayes et al., 2011).

NE serum levels are elevated in CF patients and together with increased AAT levels, they attenuate inflammation promoting wound closure in a CF *in vitro* model (Garratt et al., 2016).

CF neutrophils are less responsive to recruitment signals, have reduced bactericidal effects when recruited, exocytose high levels of degranulation molecules (NE) that lead to injury, have decreased wound repair signal levels (AAT), and have delayed apoptosis compared to healthy neutrophil functions.

### 4.2 Macrophages

CF macrophages have been found to be dysfunctional in several ways, including a decreased ability to phagocytose due to defective autophagy (Harwood et al., 2021; Badr et al., 2022; Aridgides et al., 2023). Circulating monocytes from pwCF show significantly higher baseline levels of activation compared to non-CF donors, but their ability to mount a type 1 IFN signaling response against *Pa* LPS is diminished (Gillan et al., 2023) and their response to attenuate inflammation is delayed (Slimmen et al., 2023; Wellems et al., 2023).

In addition, CF macrophages have decreased iron metabolism protein heme oxygenase-1 leading to more iron availability, which is utilized by *Pa* as a nutrient promoting *Pa* growth and biofilm formation (Hazlett et al., 2020; Harwood et al., 2021; Kircheva et al., 2024; Zhang et al., 2024). The combination of increased pro-inflammatory cytokines and an impaired phagocytotic activity and an arrest in M1 leads to over recruitment of CF neutrophils creating a positive feedback loop contributing to continuous lung damage in the CF airway.

Oz et al. demonstrated that CF macrophages positive for the CCL2 and CCL7 ligand receptor (CCR2) can drive pathogenic TGFβ signaling and sustain a pro-inflammatory environment by constitutively recruiting neutrophils. The same study found that CF mice had higher levels of M1 macrophages and neutrophils than the WT after *Pa* LPS stimulation and had more severe lung damage, collagen deposition, airway wall thickening and higher levels of TGFβ (Oz et al., 2022).

### 4.3 Lymphoid cells

Lymphoid cells express the CFTR protein and in CF, they are ineffective in clearing *Pa* at least partially due to being Th17 and Th2 cells rather than Th1, which leads to impaired antigen presentation, increased levels of immunoglobulin (Ig) E, and excessive neutrophil infiltration (Harwood et al., 2021). This Th17 and Th2 phenotype is likely in part due to increased levels of IL-17 and IL-10, which hinder antigen presentation, and of IL-6, essential for neutrophil recruitment, in pwCF (Hagner et al., 2021; Harwood et al., 2021). In an *in vivo* model mimicking CF lung disease, knockout of IL-17A led to reduced structural lung damage and decreased levels of lymphocytes, implicating IL-17A and lymphocytes as important mediators of CF lung inflammation (Hagner et al., 2021).

From a B-cell perspective, pwCF have increased lymphoid follicles and *Pa* infection leads to upregulation of the B-cell chemoattractants CXCL13, CCL19, and CCL21 (Neill et al., 2014; Polverino et al., 2019). However, there is conflicting evidence for elevation of B-cell activating factor (BAFF) in pwCF at baseline and how this affects *Pa* bacterial burden (Neill et al., 2014; Garic et al., 2019; Polverino et al., 2019).

Up to now, lymphocytes have not been shown to be involved directly in wound repair mechanisms in the CF lung.

### 4.4 Eosinophils

Eosinophils are well characterized in allergic asthma and have been described in several studies in CF. Most CF patients do not show any change in the absolute eosinophil count (AEC) in their lungs, however, the levels of eosinophil cationic protein (EPA), an eosinophil activation marker, is upregulated in the sputum and serum of pwCF (Koller et al., 1994; Liu et al., 1999). This could be due to NE mediated degranulation of eosinophils indirectly increasing EPA levels (Liu et al., 1999).

There is no evidence of correlation between *Pa* infection and eosinophil count (Albon et al., 2023), however, there is a population of pwCF that have both CF and asthma which have elevated levels of AECs, which was correlated with more symptoms and higher exacerbation numbers (Ye et al., 2022).

Overall, it does not appear that eosinophils in CF play a key role in *Pa* infection but contribute to the already dysfunctional immune response with increased levels of EPA.

Eosinophils have been found to play a role in lung injury repair. Eosinophils produce TGF-β, which is a well-known inducer of EMT (Phipps et al., 2002; Siddiqui et al., 2023). Eosinophils also express fibroblast growth factor 2, vascular endothelial growth factor, and IL-4, which have been shown in wound repair of the skin (Phipps et al., 2002). Therefore, eosinophils are potentially one of the cell types that initiate and activate remodeling and repair processes in wound healing, but more research is needed in CF eosinophils and their influence on lung epithelial repair.

**Figure 2:**
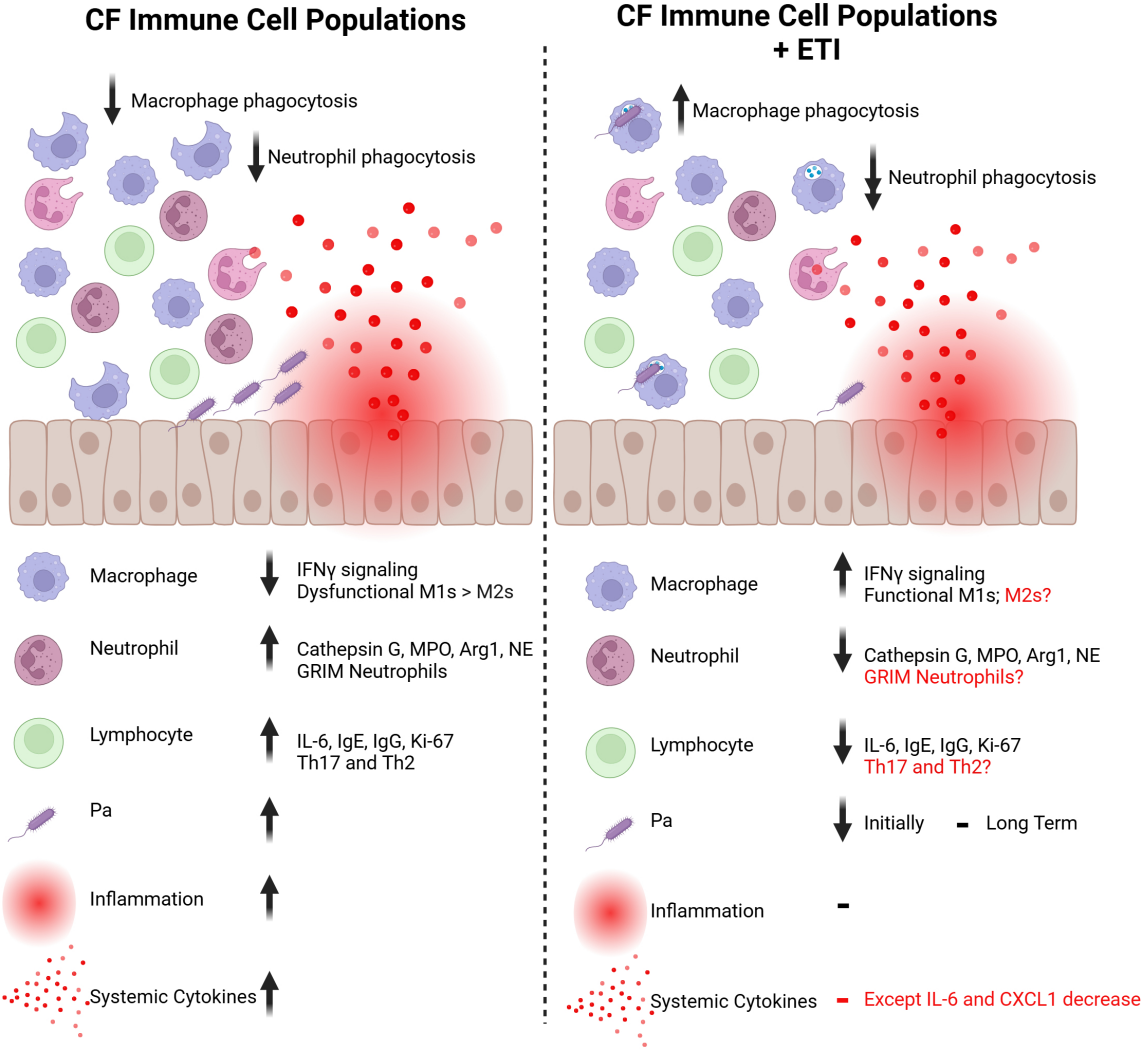
Immune cell populations in the CF bronchial epithelium before and after ETI therapy. The immune cell populations that reside in and interact with the CF bronchial epithelium have attenuated responses to injury. Macrophages and neutrophils have reduced phagocytic capabilities against bacteria like *Pa* and exacerbate inflammatory signals. With ETI therapy, CF macrophages show improved phagocytosis through an increase in IFNγ signaling. However, CF neutrophils have further decreased phagocytic capabilities. CF neutrophils tend to be over-recruited to sites of injury, which is improved with ETI initiation. Lymphocytes are also upregulated at baseline in the CF bronchial epithelium but the impact of ETI treatment on this population is largely unknown. The dysregulated immune response allows for higher levels of colonization with bacteria like *Pa*. With ETI therapy, levels of *Pa* colonization are reduced in short-term studies. However, levels of *Pa* have been shown to rebound with long-term ETI treatment. Due to this dysfunctional immune response, the CF bronchial epithelium has high levels of inflammation, resulting in higher levels of systemic cytokines. This local and systemic inflammation is not returned to baseline with ETI therapy, but improvements are seen in cytokines such as IL-6. Created in https://BioRender.com.

In summary, neutrophils and macrophages are affected in CF, and their dysfunction causes a delay in eradicating *Pa* and wound repair after *Pa* -induced injury. Lymphoid cells are also dysregulated in CF, contributing to the often-ineffective immune response to *Pa* infection. In contrast, eosinophils have not been widely implicated in CF immune responses to *Pa* but have been found to be important in lung injury repair processes in general (**Figure 2**).

## 5 Highly effective modulator therapy in CF

The highly effective modulator therapy consisting of ETI was first approved for use in the United States in 2019 to treat pwCF with at least one F508del mutation, which is the most common genetic mutation, as well as at least 177 other mutations to date (Cystic Fibrosis Foundation, 2020; Dailymed, 2020). Several studies have shown that lung function and sweat chloride significantly improve with ETI therapy (Zemanick et al., 2021). This section of the review will focus on the effect of ETI on the CF lung including the airway epithelium, wound repair, and the immune system.

### 5.1 Effects of ETI on CF bronchial epithelium

ETI can efficiently restore CFTR function in CF bronchial epithelial cells from donors with eligible mutations; ETI attenuates lipid dysregulation, such as an increased fatty acid ratio, and oxidative stress in CF bronchial epithelial cell cultures (Veltman et al., 2021). However, ETI treatment led to a two-fold increase in *de-novo* synthesis of sphingolipids in primary epithelial cell cultures in both CF and non-CF subjects, implicating that this phenomenon is a potential off-target effect of ETI therapy (Liessi et al., 2023).

### 5.2 Effects of ETI on wound repair

A 2018 study suggested that the combination of lumacaftor (VX-809) + ivacaftor (VX-770) improved wound repair rates and transepithelial resistance in primary epithelial cell cultures from CF donors with CFTR trafficking mutations (Adam et al., 2018). This effect was also observed in the presence of *Pa* exoproducts. In another study, Laselva et al. demonstrated that ETI-treated bronchial epithelial cells that overexpress the F508del mutation exhibit accelerated wound repair relative to vehicle treated controls (Laselva and Conese, 2022), whereas Easter et al. did not find any attenuation in the pro-inflammatory response nor reduction in cellular senescence in the bronchial epithelium (Easter et al., 2024). However, these studies did not explore the effect of ETI on any wound repair specific markers in CF airways (**Figure 1**).

### 5.3 Effects of ETI on *Pa* infection

Introduction of ETI was presumed to lead to eradication of *Pa* in the CF lung, given the rapid decrease in *Pa* bacterial burden found with initiation of Ivacaftor and only modest increase in *Pa* one year post-Ivacaftor initiation (Hisert et al., 2017). Early studies assessing the effect of *Pa* burden on pwCF post ETI initiation have demonstrated a modest decrease in positive *Pa* cultures; however, *Pa* is not eradicated in pwCF on ETI therapy for more than one year (Gushue et al., 2023; Burgel et al., 2024; Dittrich et al., 2024; Szabo et al., 2024). Recent reports have shown that reduction or eradication of *Pa* is more likely in pwCF, who are younger and colonized for a shorter period of time, when compared to older pwCF, who are colonized for longer and with a multi-drug resistant *Pa* (Dittrich et al., 2024; Szabo et al., 2024).

Taken this together, *Pa* colonization and recurrent infections are still evident in the post-ETI era. Analysis of the *Pa* genome pre- and post- ETI initiation demonstrates strains do not change post-ETI initiation, but *Pa* has the ability to adapt in this new environment showing mutations in iron metabolism, alginate production, and biofilm formation (Armbruster et al., 2024; Ledger et al., 2024).

### 5.4 Effects of ETI on immune cells

Although lung function and mucociliary clearance improves in the majority with ETI therapy, the effect of ETI on the innate immune system and systemic inflammation is controversial (**Figure 2**). Although there seems to be a trend of ETI-mediated decrease in pro-inflammatory markers, those levels do not reach a non-CF baseline level (Casey et al., 2023; Schmidt et al., 2023; Wang et al., 2023; Westholter et al., 2023; Jarosz-Griffiths et al., 2024). Most studies have found decreases in pro-inflammatory markers including IL-6, MMP9, and CXCL1, but no differences in IL-8 levels, which is the key cytokine mediating neutrophil recruitment in the CF lung.

The effect of ETI on neutrophils is an ongoing area of research with one study observing no differences in neutrophil activation marker baseline levels between pwCF before ETI and 6 months post initiation (Schmidt et al., 2023). Furthermore, ROS production and chemotactic activity did not change post ETI, and the phagocytotic capacity decreased down post ETI initiation, in contrary to the expected improvement.

Neutrophil cytokine secretion of IL-1β, TNF, and IL-6 decreased with ETI therapy, along with caspase-1 levels (Jarosz-Griffiths et al., 2024) and the number of sputum neutrophils remained reduced from 3 months to 1 year post ETI initiation in addition to decreases in NE, AAT, and systemic decrease in IL-8 (Casey et al., 2023).

These data would indicate that although neutrophils secrete less proinflammatory mediators, their ability to be activated and phagocytose is not fully restored by ETI treatment. These data also highlight the importance of further studies, especially longitudinal studies to determine the long-term effects of ETI on the neutrophil and other immune cell populations at different stages of CF lung disease severity.

Some studies have shown that in the presence of ETI therapy and either *Pa* or *Burkolderia cepacia* complex infection, CF macrophage phagocytosis and killing improved but cytokine secretion was not affected (Aridgides et al., 2023; Zhang et al., 2023). These papers did not address whether the total number of macrophages was impacted by ETI but did show that ETI enhanced the M1 phenotype with little benefit for M2 macrophages, which are involved in injury repair and resolution of inflammation. Additionally, it has been shown that the stunted IFN signaling in macrophages is rescued with ETI therapy (Gillan et al., 2023).

The current literature is controversial in regard to the benefits of modulator therapy on lymphoid cells. One report did not mention differences in circulating T cells with mono- or dual modulator therapy, whereas another study observed increased levels post ETI therapy (Eschenhagen et al., 2023; Westholter et al., 2023). Westholter et al. noted decreased IL-6 serum levels 6 months post ETI initiation and Eschenhagen et al. reported decreases in IgE, IgG, and Ki-67 levels post ETI initiation (Eschenhagen et al., 2023). In the presence of *Pa* infection, some reports show decreased levels of regulatory T cells (Tregs) (Westholter et al., 2023).

There is very little known regarding the effect of ETI therapy on the eosinophil population, and eosinophils are not key mediator cells in *Pa* infections. However, one recent study showed that ETI led to a decrease in IgE levels but did not affect eosinophil count in a study population of 108 pwCF (Mehta et al., 2023).

## 6 Future directions and discussion

### 6.1 Treatments to benefit the CF lung

Although modulator therapy has greatly increased the life expectancy and quality of life in most pwCF, there are still patients not eligible for HEMT and others who experience a limited impact on various cell populations in the CF lung. Therefore, continued research is needed to better characterize short- and long-term effects of ETI that takes the severity of disease, as well as severity of lung injury, into consideration.

#### 6.1.1 CFTR modulators

Current studies in the field of modulators include small molecule screening to target nonsense-mediated decay of the CFTR transcript due to stop codon mutations (Sharma et al., 2021; Kim et al., 2022; Chen et al., 2023). There are also ongoing clinical trials for mRNA correction therapy (Rowe et al., 2023).

Additionally, to add to the current modulators, compounds able to stabilize or amplify the CFTR protein are another area of research that is being done in this field to increase the half-life of the CFTR protein (Donaldson et al., 2017; Bengtson et al., 2022).

#### 6.1.2 Anti-inflammatory treatments

To combat the chronic inflammation found in pwCF, there are a few avenues being researched as potential therapeutic targets for regulating the inflammatory axis. TNF signaling, more specifically the LTa-TNFN2 axis, has been implicated as a therapeutic target given the protective role that Etanercept, a molecule that increases oxidative stress resistance, exerts on epithelial cells *in vitro* when challenged by H2O2 (Checa et al., 2024).

IL-22 signaling is also being considered as a therapeutic target given its role as an integral molecule to prevent *Pa*-mediated lung damage. PwCF have lower levels of IL-22 which is associated with increased levels of serine protease-3 (Guillon et al., 2019). Therefore, either supplementation with IL-22 and/or reinstating the protease to anti-protease ratio will be pivotal for resolving chronic inflammation in CF.

Other studies explore ways to improve inflammation in CF by acting on specific immune populations. One such study found that the compound Tanshinone IIA promotes decreased inflammation and tissue repair by inducing neutrophil reverse migration and induced apoptosis in an *in vivo* CFTR KO zebrafish model, which could also be a potential therapeutic target for the delayed apoptosis of the CF neutrophil (Bernut et al., 2020). Another potential therapeutic target could be formyl peptide receptor 1 (FPR-1) which regulates neutrophil function and is expressed on their cell surface. Mice deficient in this protein were protected from lung injury via a bleomycin model (Leslie et al., 2020).

The use of IL-6-elafin (a serine protease inhibitor) genetically modified macrophages have been found to better fight *Pa* infections and were found to increase epithelial cells proliferation and tissue repair in a murine model (Kheir et al., 2022). Finally, Cappiello et al. found esculentin (Esc) (1-21) and Esc(1-21)-1-c, both antimicrobial peptides, detoxify LPS shown through decreases in IL-6 and cyclooxygenase-2 and further lead to cell migration in epithelial cells (Cappiello et al., 2021). Suggesting that these molecules could also be therapeutic targets for chronic infections such as *Pa* and assist the immune cell population.

Another avenue to potentially promote resolution of chronic inflammation is to elucidate the role of extracellular vesicles (EVs) in the context of *Pa* infection. EVs have been found to be particles that contain miRNA, ECM proteins, cytokines, and much more (Useckaite et al., 2020; Sarkar et al., 2024). They have been implicated in several disease states including CF. Recently, a study found that EVs secreted by bronchial epithelial cells were able to reduce *Pa* burden and inflammation in CF mouse lungs (Sarkar et al., 2024). Given this, further research regarding the role that EVs play in these mechanisms will be crucial to finding therapeutic targets for both chronic inflammation and chronic infection with *Pa*.

One study explored treatment with the senolytic cocktail Dasatinib + Quercetin (D+Q) that not only decreased cellular senescence markers in a *Cftr-/-* knockout rat trachea model but also decreased pro-inflammatory cytokine secretion and significantly improved mucociliary clearance (Easter et al., 2024). This same study by Easter et al. also explored the mechanisms governing this increase in senescent cells and found that fibroblast growth factor receptor inhibition attenuated senescence markers and improved mucociliary clearance.

Continued research in this field will be vital to uncovering ways to control the chronic inflammation that exacerbates respiratory complications found in CF.

#### 6.1.3 Treatment strategies to promote tissue repair

There are not many studies on the promotion of tissue repair in the CF lung, but one such study found that in addition to being preventative for inflammation, IL-22 was also found to lead to higher wound-closure rates *in vitro* (Guillon et al., 2019). Leading to the possibility of IL-22 treatment being a functional treatment for multiple aspects in tissue repair and inflammation.

Other interesting discoveries include the use of electro-sprayed mesenchymal stromal cell extracellular matrix nanoparticles to accelerate cellular wound healing and also reduce *Pa* growth (Wandling et al., 2023). Another study found that antipseudomonal peptides esculentin-1a(1-21)NH2 and Esc(1-21)-1c were found to counteract the inhibitory effect of *Pa* -LPS on wound healing which in turn enhanced IL-8 production and MMP9 activity (Cappiello et al., 2019).

### 6.2 Advancements in treatments to target *Pa* infection in the CF lung

*Pa* is notoriously difficult to eradicate due to adaptive systems that can evade antibiotics. These include biofilm-mediated resistance, hypermutation of antibiotic targets, and formation of multidrug-resistant cells (Mulcahy et al., 2010; Breidenstein et al., 2011). The current treatment regimen for *Pa* in the CF lung is to attempt early eradication with inhaled antibiotics, tobramycin or tobramycin/ciprofloxacin (Mogayzel et al., 2014). This approach aims to attenuate *Pa*-mediated airway remodeling, but has been not very successful for most pwCF. Most develop *Pa* colonization and chronic suppressive therapy with inhaled antibiotics, such as tobramycin, colistin, or aztreonam lysine, becomes the mainstay of treatment (Smith and Rowbotham, 2022).

While the development of novel antimicrobials is desperately under explored, some promising and innovative examples are currently in the pipeline. This section is not exhaustive of all new potential *Pa* treatments; however, it highlights some of the areas that are generating the most interest currently. Treatments targeting *Pa* colonization and/or infection will indirectly improve the processes of wound repair, as *Pa* possesses many virulence factors that directly affect damage and repair (as reviewed in Section 3). Lowering the levels of *Pa* in the lung would also indirectly affect immune function, as *Pa* is highly pro-inflammatory.

#### 6.2.1 Conventional antibiotics

Avibactam, vaborbactam, and relebactam are among the new β-lactam–β-lactamase inhibitors which have shown promising results when combined with other conventional pseudomonal antibiotics (Yahav et al., 2020). Cefiderocol is a novel siderophore cephalosporin with activity against a wide spectrum of Gram-negative micro-organisms, including multidrug-resistant *Pa* infections (Hackel et al., 2018; Meschiari et al., 2021). Plazomicin is a parenteral aminoglycoside recently approved by the FDA for the management of complicated urinary tract infections and pyelonephritis caused by susceptible organisms, including *Pa* (Losito et al., 2022). While it does not seem to be superior to other aminoglycosides such as amikacin, gentamicin, and tobramycin against *Pa*, it does give health-care providers a new tool.

#### 6.2.2 Anti-biofilm and anti-quorum sensing

Biofilms and bacterial communications are a source of antibiotic resistance; therefore, some groups are developing strategies to directly target these systems. One example is a low molecular weight alginate oligomer, known as OligoG. *In vitro* studies have demonstrated it was able to inhibit *Pa* mucoid biofilm formation and disrupt established mucoid biofilms. It was also able to potentiate the effects of other antimicrobials by increasing diffusion within biofilms (Powell et al., 2018). OligoG has now entered phase 2b clinical trials to assess safety, efficacy and tolerability in adult pwCF (2019). Another example of biofilm disruption is PLG0206, an engineered cationic antimicrobial peptide. Initial studies have demonstrated broad-spectrum activity and the capacity to significantly inhibit *in vitro Pa* biofilm growth on airway epithelial cells (Lashua et al., 2016), which ultimately can attenuate chronic inflammation and aberrant wound repair.

#### 6.2.3 Phage therapy

Phage therapy has been an increasingly popular alternative for antibiotic treatment, especially for patients who have been unresponsive to conventional antibiotics. Due to limited production and distribution, most cases of successful bacteriophage usage are described in individual case reports, however, few examples are promising. A personalized aerosolized bacteriophage treatment was successfully used to treat a patient with chronic lung infection caused by multidrug-resistant (MDR) *Pa* (Kohler et al., 2023) resulting in clinical improvement and progressive clearance of lung consolidations. Another case described by Law et al. used bacteriophage therapy in a 26-year-old cystic fibrosis (CF) patient awaiting lung transplantation for pneumonia treatment. The patient did not have recurrence of *Pa* pneumonia and CF exacerbations within 100 days following the end of therapy and underwent successful bilateral lung transplantation 9 months later (Law et al., 2019).

There are several clinical trials currently underway to study the safety and efficacy of using single and multi-phage treatments to target *Pa* in pwCF (2020; Tamma et al., 2022; Ling et al., 2023). While these treatments and trials are exciting, there still are many unanswered questions regarding phage therapy, specifically the effect of phages on wound repair mechanisms in the chronically infected lung.

#### 6.2.4 Vaccines

Finally, a true breakthrough discovery for *Pa* infection prevention would be an effective vaccine, the hunt for which has been underway for nearly 50 years. An important challenge for obtaining a good vaccine is the identification of ideal antigens. Antigen variability, plasticity of virulence factor expression during acute to chronic infection and bacterial localization are a challenge (Hoggarth et al., 2019; Sainz-Mejias et al., 2020; Lopez-Siles et al., 2021). However, if successfully developed, a vaccine against *Pa* for at-risk patients could reduce the prevalence of infections, the overall burden of disease, and the use of antibiotic treatment. Four vaccines have entered phase III trials with some showing promising results, but no anti- *Pa* vaccine has yet been approved. As discussed for other anti- *Pa* treatments, it remains to be studied whether vaccines would have direct effects on wound repair mechanisms.

## 7 Conclusion

In conclusion, the wound repair mechanisms and immune cell function are both altered in the CF lung, which is further exacerbated by many virulence factors of *Pa*. There are several cellular mechanisms we discussed that could use more exploration, such as specific cell signaling pathways altered in dysfunctional wound repair, or the possible roles that eosinophils can play in wound repair.

The FDA approval of HEMT has changed the clinical landscape of care for over 90% of pwCF. We discussed the current evidence that ETI is improving metrics in inflammation and bacterial burden, but many of these metrics are not returned to baseline states when compared to non-CF. Notably, *Pa* is not always eradicated from the CF lung after ETI initiation, and the same *Pa* strains have been observed in pwCF pre- and post-ETI initiation. In addition, *Pa* has impressive versatility and the ability to adapt quickly in new environments, raising concerns on the continued effects of *Pa* on repair mechanisms in the ETI-treated CF bronchial epithelium.

ETI treatment has also not been well explored in the context of cell signaling mechanisms, leaving many questions on how this treatment will change the CF lung’s ability to “repair itself”. Additionally, we explored other potential avenues of treatment for pwCF, ranging from anti-inflammatories to phage therapy. These studies demonstrate the need for further research into the cellular mechanisms attributing to CF disease states, highlight the currently evolving landscape of treatment with ETI, and shed light on treatments that could be available in the future for pwCF, regardless of their CF mutations.
